# Reproducible determination of transpulmonary pressures

**DOI:** 10.1016/j.mex.2022.101696

**Published:** 2022-04-09

**Authors:** G.R.A. De Meyer, S.G. Morrison, V. Saldien, P.G. Jorens, T. Schepens

**Affiliations:** a Antwerp University Hospital, Edegem, Belgium; b University of Antwerp, Antwerp, Belgium

**Keywords:** R, Oesophageal balloon catheter, Respiratory monitor, Fluxmed, Data Extraction

## Abstract

Oesophageal pressures, as measured in an oesophageal balloon catheter, are a validated substitute for pleural pressures.

Transpulmonary pressures, indispensable to improve our understanding of ventilatory physiology, are therefore typically calculated as the difference between airway and oesophageal pressures.

The oesophageal pressure signal, however, features a superimposed oscillation due to cardiac motion, not representative for pleural pressure. Additionally, oesophageal contractions or surgical manipulation can alter the signal. In practice, transpulmonary pressures are therefore manually determined from the pressure-time graphic by visual inspection of the waves and averaging a limited number of samples.

We suggest an approach to extract the end-expiratory transpulmonary pressure from the raw monitoring data.

• Our approach reproducibly determines end-expiratory transpulmonary pressures at a given level of set positive end-expiratory pressure at the ventilator.
• Our approach ignores surgical disturbance and cardiac oscillations in the oesophageal pressure signal.

## Graphical abstract

**Figure:**
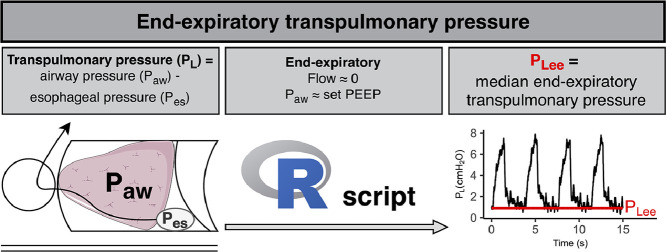

## Data extraction

Specifications table

**Table:** 

Subject Area:	Medicine and Dentistry
More specific subject area:	Mechanical ventilation, artificial ventilation
Method name:	Reproducible determination of transpulmonary pressures
Name and reference of original method:	• [Oesophagusdruk en longelasticiteit] oesophageal pressure and lung elasticity – Buytendijk [1]. • A Comparison of oesophageal and Intrapleural Pressure in Man – Cherniack et al. [2]. • A method of assessing the mechanical properties of lungs and air-passages - Dornhorst and Leathart [3]. • The application of oesophageal pressure measurement in patients with respiratory failure – Akoumianaki et al. [4].
Resource availability:	• Respiratory monitor with software e.g., FluxMed GrT respiratory monitor with FluxView and FluxReview software, MBMED, Argentina, mbmed.com or BIOPAC MP36R system with AcqKnowledge software, biopac.com. • Compatible flow sensor. • Oesophageal balloon catheter e.g., AVEA® SmartCath 8Fr oesophageal balloon catheter, Vyaire Medical, USA, vyaire.com. • R software environment for statistical computing, r-project.org. • RStudio integrated development environment for R, rstudio.com. • Tidyverse R package, cran.r-project.org. • A copy of the example files, directories and R script, github.com/gregorydm/PLee.

## Method details

### Requirements

**Fig. 1:**
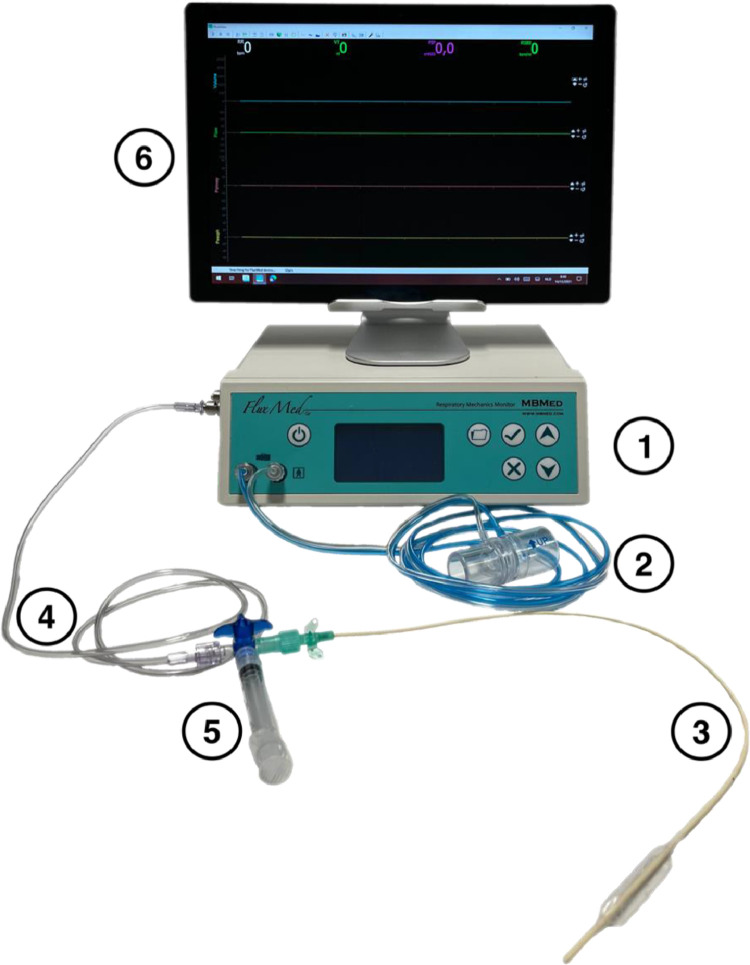
Respiratory monitor with disposables. Numbers correspond to the text under ‘Requirements’.

The following resources should be obtained (Fig. 1):

1. A respiratory monitor with a raw data export option to simultaneously record time, airway pressure, airway flow and oesophageal pressure.
  Our setup used the FluxMed GrT respiratory monitor with the FluxView (version 1.33i) and FluxReview (version 1.33i) software packages (MBMED, Argentina).
2. A pressure and/or flow sensor in the breathing circuit that is compatible with the respiratory monitor.
  We used MBMED's disposable adult flow sensor (MBMED, Argentina).
3. An oesophageal balloon catheter, compatible with the respiratory monitor, to measure the oesophageal pressure.
  We opted for the AVEA® SmartCath 8Fr oesophageal balloon catheter (Vyaire Medical, USA).
4. A non-compliant pressure extension line with compatible connections.
  These lines are often available as an accessory to pressure transducer kits (e.g., TruWave, Edward Lifesciences, USA).
5. A three-way tap with a syringe to inflate the oesophageal balloon.
6. A computer to run the different software packages, to save the raw data on and to execute the purpose-made script.
7. The open source R language & environment for statistical computing [5]. We used version 3.6.3 "Holding the Windsock".
8. The GNU3-licensed RStudio integrated development environment for R [6]. We used version 1.4.
9. The open source tidyverse collection of R packages [7]. We used version 1.3.
10. A copy of the purpose-made R script and directory structure as provided in the supplemental materials.

### Measurement setup

The open-label, single-centre, repeated-measures clinical trial in which we applied this method, included anaesthetized and mechanically ventilated participants (clinicaltrials.gov NCT04900714). Healthy, non-obese patients without a history of respiratory illness, who were scheduled for elective pelvic laparoscopic surgery in the Trendelenburg position were eligible for inclusion. The intervention was a recruitment manoeuvre followed by a stepwise reduction in positive end-expiratory pressure (PEEP) from 15 to 10 to 5 cmH_2_O. The abdominal inflation pressure was 15 mmHg (approximately 18 cmH_2_O) in all patients. The operating table slope remained unchanged between the different PEEP levels (IQR 22°– 25° Trendelenburg).

**Fig. 2:**
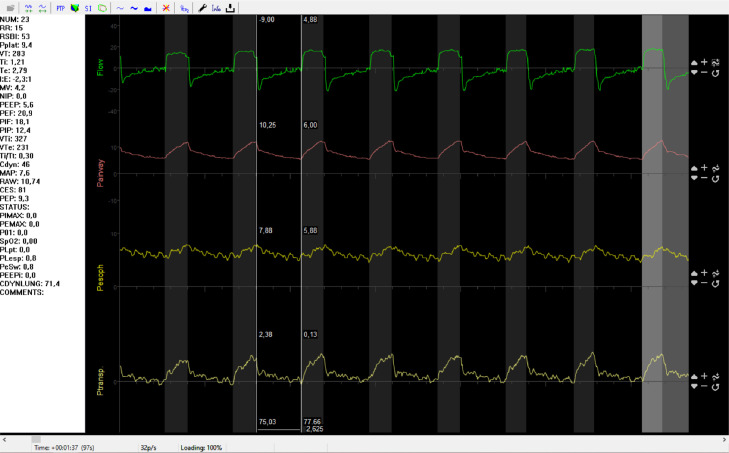
Screenshot from the FluxReview software illustrating from top to bottom flow(time), P_aw_(time), P_es_(time) and P_L_(time) curves. Note the cardiac oscillations present in the P_es_(time) wave.

The methodology may be adapted for awake, spontaneously breathing participants using a mask with a mounted flow-sensor.

Power on and calibrate the respiratory monitor. Connect the computer and launch the acquisition software. Place the flow sensor in the breathing circuit and connect to the respiratory monitor after successful calibration. Connect the hard pressure line with a three-way tap and syringe to the oesophageal or auxiliary pressure port on the respiratory monitor. Initiate recording on the respiratory monitor and critically assess the waves. The signals should reflect the settings of the ventilator and no significant leaks ought to be present.

Position the oesophageal catheter carefully. Its tip should sit in the distal third of the oesophagus. Akoumianaki et al. detail the steps to adequately position the catheter [8]. In most adults, a normal depth of insertion is between 30 and 40 cm.

Connect the balloon to the pressure line and inflate using the syringe and three-way tap. Be aware that too much volume in the balloon leads to falsely elevated absolute oesophageal pressure (P_es_) measurements, while too little volume results in an underestimation of changes in oesophageal pressure (∆P_es_). Mojoli et al. describe an *in vivo* calibration method to determine the optimal filling volume in mechanically ventilated patients [9]. According to the manufacturers recommendations, we first inflated the balloon with 2 ml air and then extracted 1 ml, resulting in a filling volume of 1 ml.

Positioning of the oesophageal catheter must be verified. In short, cardiac oscillations should be apparent (Fig. 2) and the ratio of change in oesophageal pressure to change in airway pressure (∆P_es_/∆P_aw_ ratio), as noted during an expiratory occlusion manoeuvre with chest compression, should be between 0.8 and 1.2 [10] A detailed guide to verify correct oesophageal catheter placement can be found in the review by Akoumianaki et al. [8]

Note the time at which the experiment is started in the Case Report Form (CRF). It will be needed to determine the representative part of the recording.

To improve the quality of the calculation, disturbance of the signal should be avoided as far as possible, while the duration of a stable signal recording should be maximized. In our setup, surgery was ongoing, and signals were recorded for approximately 7 min at different protocol-determined levels of PEEP.

### Raw signal data

We use a standardized and generic project directory structure (see supplemental materials). 

Each study participant has a dedicated folder to hold its raw data. The name of the folder refers to the subject's identification (ID). Use the software from the respiratory monitor to export the raw data and save it in the folder with the corresponding ID. Make sure to choose a file structure that is convenient to import into R, such as tab separated values (tsv) or comma separated values (csv). Associate the participant's ID with the file name of the export.

The FluxReview software exports raw data as tab separated files. The software provides two export options - signals and parameters, where signals refer to the time series generated by the sensors. It contains the columns Time, Flow, Volume, P_aw_ (airway pressure), P_L_ (transpulmonary pressure), P_ga_ (gastric pressure), P_tdiaf_ (transdiafragmatic pressure) and CO_2_ (expired CO_2_). A data point is available for each column every 3.9 ms, reflecting FluxMeds sampling frequency of 256 Hz (Table 1).

**Table 1:** Example of FluxView raw data file.

FluxView - FluxMed acquisition system								
Signal file								
Serial Number								
SW version: 1.33i-OX-CO2 | File version:1.15								
Sampling rate: 256 Hz								
Time	Flow	Volume	P_aw_	P_es_	P_L_	P_ga_	P_tdiaf_	CO_2_
Sec	l/min	ml	cmH_2_O	cmH_2_O	cmH_2_O	cmH_2_O	cmH_2_O	mmHg
0	0	0	0	0	0	0	0	0
0,0039063	−5,6	0	6,1	0	6,1	0	0	0
0,0078125	−5,1	−1	6,3	0	6,3	0	0	0
0,0117188	−4,8	−1	6,4	0	6,4	0	0	0
0,015625	−4,4	−1	6,4	0	6,4	0	0	0
0,0195313	−4,5	−1	6,1	0	6,1	0	0	0
0,0234375	−5	−1	6,3	0	6,3	0	0	0
0,0273438	−5,4	−1	6,4	0	6,4	0	0	0
0,03125	−5,6	−1	6,4	0	6,4	0	0	0
0,0351563	−5,6	−2	6,1	0	6,1	0	0	0
0,0390625	−5,5	−2	6,1	0	6,1	0	0	0
0,0429688	−5	−2	6,3	0	6,3	0	0	0
0046875	−4,5	−2	6,3	0	6,3	0	0	0

Note that P_es_, P_ga_ & CO_2_ were not connected. P_L_ = P_aw_–P_es_.

SW = software, Hz = Hertz, P_aw_ = airway pressure, P_es_ = oesophageal pressure, P_L_ = transpulmonary pressure, P_ga_ = gastric pressure, P_tdiaf_ = transdiafragmatic pressure = P_es_ - P_ga_, CO_2_ = carbon dioxide, Sec = seconds, l/min = litres per minute, ml = millilitres, cmH_2_O = centimetres of water pressure, mmHg = millimetres of mercury pressure.

### Calculation steps and rationale

The standard clinical practice of visually inspecting transpulmonary pressure-time plots, P_L_(t), is labour intensive and therefore limits the amount of data points that can be used to calculate a representative mean or median transpulmonary pressure during the period of interest. Additionally, this method is prone to inter-observer bias. However, this approach has the advantage that a trained clinician can judge the quality of the recording while only taking samples at representative points and ignoring, for instance, the effects of oesophageal contractions or patient manipulation.

**Fig. 3:**
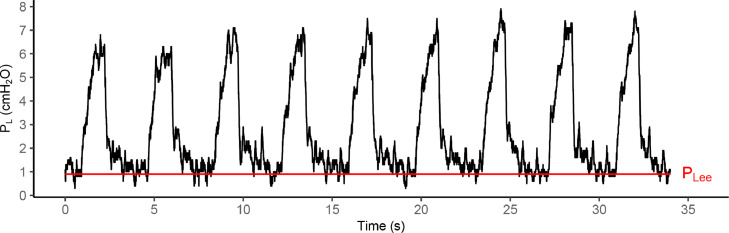
Graphical illustration of calculated median end-expiratory transpulmonary pressure (red line) in reference to the raw transpulmonary pressure (P_L_) - time wave. cmH_2_O: centimetres of water pressure; P_Lee_: end-expiratory transpulmonary pressure; s: seconds.

We designed a method of data extraction, using all representative data points, with the aim of improving reproducibility (Fig. 3).

We identified four important steps to obtain the representative end-expiratory transpulmonary pressure from the raw data recording.

1. Remove all data points recorded before the start or after the end of the experiment.
2. Filter for end-expiratory values only. At end-expiration:
  a. the recorded airway pressures should be close to the set positive end-expiratory pressure (PEEP).
    We used a margin of ±1 cmH_2_O around set PEEP. For example, if PEEP was set at 15 cmH_2_O, the airway pressures in the raw data were filtered for values between 14 and 16 cmH_2_O to account for variation in the measurement.
  b. the recorded flow should approximately be zero.
    For the flow, we used a margin of ±10 L/min around zero as some noise on the recording may be present.
3. Remove all non-sense data points from the end-expiratory transpulmonary pressure column. According to Tukey's rule, all outliers smaller than *Q1 - 1.5 x IQR* or larger than *Q3 + 1.5 x IQR* were removed [11]. Alternatively, other techniques of outlier detection may be considered. However, Tukey's rule is known to be robust and is easily applicable for the removal of multiple outliers [12].
4. Calculate a summary statistic that is representative for the end-expiratory pressure during the period of interest. We decided to calculate the median, as this is a robust measure of central tendency least sensitive to extreme values.

As this approach would still be manually labour intensive, we programmed the steps into a purpose-build script in R that iterates over all raw data files in the selected directory. The interventions in the clinical trial for which the script was designed were high (15 cmH_2_O), medium (10 cmH_2_O) or low (5 cmH_2_O) PEEP. These PEEP steps are provided as inputs for the function in the script. Note that the function removes all rows outside set PEEP ± 1cmH_2_O. We opted to take set PEEP, instead of automatic (read) PEEP, as an input for the function. Since P_L__ee_ mainly depends on set PEEP, this reflects the most common use in research cases. The script produces an output table with 3 columns: ID, set PEEP and median P_Lee_, the last being the median end-expiratory transpulmonary pressure at the indicated level of PEEP.

### Computer setup

Download and install R from https://cran.r-project.org/mirrors.html. Subsequently, download and install RStudio from https://www.rstudio.com/products/rstudio. Launch RStudio and install the Tidyverse package by executing *install.packages("tidyverse")* in the R console on the lower left.

### The script

The R script provided will calculate the median end-expiratory transpulmonary pressure at the inputted level of PEEP.

Download the project template containing the directory structure, the R script and sample data from the supplemental materials. Note that all directories in the R script are defined relative to the *ProjectName* parent folder.

Open *ProjectName.Rproj*. RStudio will launch. Using the files pane on the lower right, navigate to *3-DataCollection* and open the R script named *Calculate median PLee.R*.

First load the Tidyverse package. Then import the CRF data. An example of a CRF export, containing minimal data (*CRF.csv)* is included in the *3-Data* subdirectory. It contains two columns: *id* is the participant's identification code and *datastart* refers to the time at which the experiment started in seconds, after initiation of the respiratory monitor recording.

As we want to calculate the median end-expiratory transpulmonary pressure per PEEP level for multiple participants, it is convenient to first define an R function and then apply this function to the data of each participant.

The function has two input variables - *id* & *start -* and performs several steps:

1. Import the raw monitoring data “signals.txt”.
2. Remove all data before start of the experiment.
3. Calculate the median end-expiratory transpulmonary pressure at the inputted level of PEEP.
4. Output the calculations as a table containing the columns *id, setPEEP* and median*P_Lee_* for the selected participant and level of PEEP.

The script has been annotated to indicate the details of each step in the function.

After defining the function, we only need to apply it to each participant-set PEEP combination using a loop. A table with the median P_Lee_ at each PEEP level for all participants is constructed (Table 2).

**Table 2:** Script example output.

id	setPEEP	medianP_Lee_
Participant001	15	−1
Participant001	10	−1.3
Participant001	5	−3.1
Participant002	15	1
Participant002	10	0.4
Participant002	5	−0.9
Participant003	15	1.9
Participant003	10	0.9
Participant003	5	0
Participant004	15	0.9
Participant004	10	−1.8
Participant004	5	−4.3
Participant005	15	5.1
Participant005	10	3.6
Participant005	5	2.6

id: identification; set PEEP: set level of positive end-expiratory pressure (cmH_2_O); medianP_Lee_: median end-expiratory transpulmonary pressure (cmH_2_O).

### Method validation

The end-expiratory transpulmonary pressures (P_Lee_) obtained by routine clinical practice of visual inspection of the waves were compared to the values obtained with the script for 5 participants. Visual inspection of the transpulmonary pressure waveform was performed by an experienced clinician (TS). At each level of PEEP, 4 representative end-expiratory transpulmonary pressures were read and averaged. A total of 15 paired values, 3 different levels of PEEP in 5 participants, were used to assess correlation and bias between the visually determined P_Lee_ and the calculated P_Lee_.

**Fig. 4:**
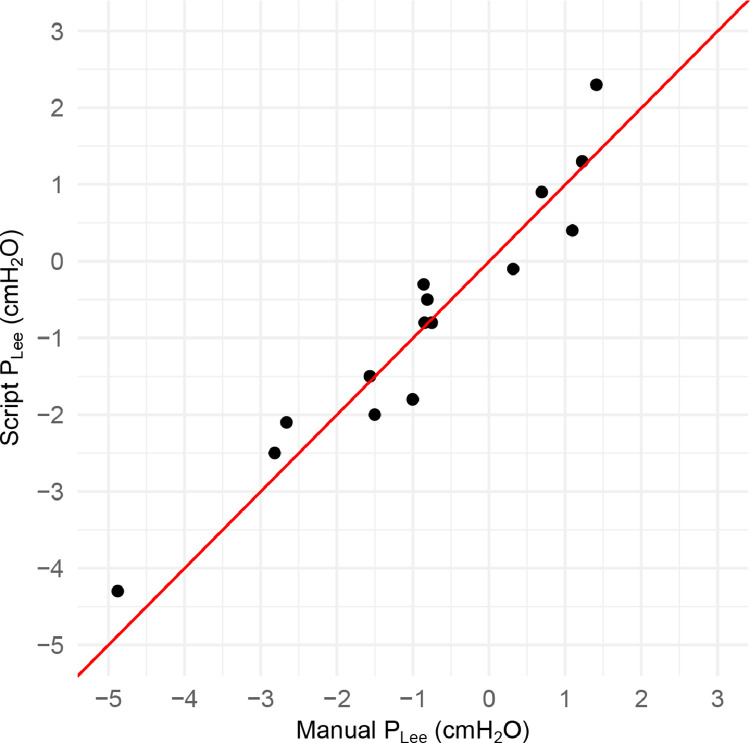
Correlation plot comparing the manual determination of the end-expiratory transpulmonary pressure (P_Lee_) with the scripted calculation method. cmH_2_O: centimetres of water pressure.

Both methods correlated significantly (Pearson's correlation coefficient of 0.96 with 95% CI 0.87–0.99, *p* < 0.001) as represented in the correlation plot (Fig. 4).

**Fig. 5:**
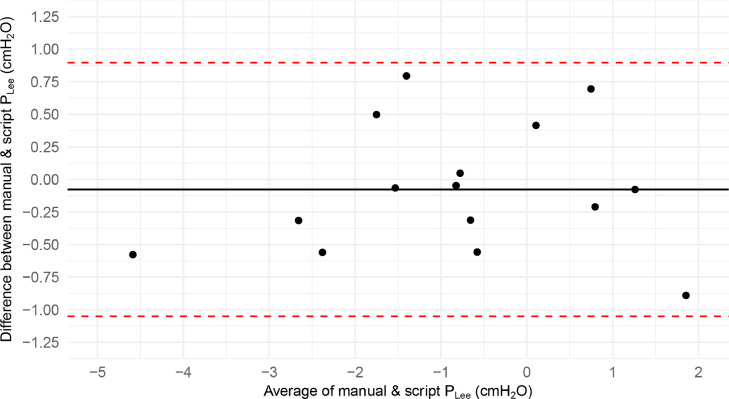
Bland-Altman plot comparing the manual determination of the end-expiratory transpulmonary pressure (P_Lee_) with the scripted calculation method. cmH_2_O: centimetres of water pressure.

A Bland-Altman plot further illustrates excellent concordance (Fig. 5). The bias between both methods was 0.08 cmH_2_O (SD 0.5 cmH_2_O). All values fall within ± 1.96 times the standard deviation. From a clinical point of view, this bias and spread are neglectable.

### Additional usages & possible refinements

Contrary to its effects on oesophageal pressure, cardiac motion does not influence pleural pressures. If the heart rate were recorded along with the oesophageal pressure, the superimposed cardiac oscillations could be filtered from the oesophageal pressure-time signal, Pes(t) using Fourier's analysis.

The method can be modified to extract other phases of the transpulmonary or oesophageal pressure wave by altering the filter criteria. For example, median transpulmonary plateau pressures (P_Lplat_) could be extracted using the criteria "P_aw_ > set PEEP + 2 cmH_2_O & flow around zero (e.g., between −10 and +10 L/min)”. Transpulmonary driving pressures could then be calculated as P_Lplat_ - P_Lee_.

## Appendix

A repository containing the script with example data.

Download the repository as a .zip file to run the script on the example data: https://github.com/gregorydm/PLee/archive/refs/heads/main.zip

Ventilator protocol

Image, application 1
