# Supplementary material for: Reproducible determination of transpulmonary pressures
Source: MethodsX. 2022 Apr 9;9:101696. doi: 10.1016/j.mex.2022.101696 (PMC9039861; doi:10.1016/j.mex.2022.101696)
Supplement: Supplementary file 1 [file mmc1.docx]

**Supplemental material: detailled ventilator protocol OptiPEEP**

Standard monitors were applied (ECG, non-invasive blood pressure and peripheral arterial saturation) and all participants were pre-oxygenated before induction of total intravenous anesthesia. This followed a standardized technique using a target-controlled infusion of propofol (Marsh model 3-6μg/l), sufentanil (0.2μg/kg) and rocuronium (0.6mg/kg). Neuromuscular blockade was maintained with additional boluses of rocuronium to ensure the train of four ratio was ≤ 1 (MechanoSensor, GE healthcare, Chicago, IL, USA). The trachea was intubated with an endotracheal tube (ETT, 7.5mm for women, 8.5mm for men, Shiley TaperGuard, Covidien, Tullamore, Ireland) and instrumental dead space reduced using an elbow piece and pediatric heat and moisture exchanger (HME; Gibeck Humid-Vent Pedi straight, Teleflex, Wayne, PA, USA). The pressure-and-flow sensor (FluxMed, Buenos-Aires, Argentina) was mounted between the ETT and the HME, and volumetric capnography (Capnostat 5, Philips, The Netherlands) measured between the HME and the Y-piece. Mechanical ventilation was initiated in volume control mode with a tidal volume of 6 ml/kg of ideal body weight (IBW), an initial PEEP of 5 cmH_2_O, a frequency of 15 breaths per minute, an inspiratory:expiratory (I:E) ratio of 1:2, an inspiratory pause of 15% and an FiO_2_ of 0.4. Minimum fresh gas flow was set at or above the minute volume (MV) to ensure a consistent FiO_2_ and to prevent rebreathing within the anesthesia circle circuit. End-tidal CO_2_ (ETCO_2_) was maintained between 35-50 mmHg by adjusting the ventilator settings as follows: the respiratory rate was increased to a maximum of 25 breaths per minute, ensuring expiration was complete by reading the expiratory flow curve; if insufficient, the tidal volume was increased to 8 ml/kg IBW.
